# Statins, Vitamin D, and Cardiovascular Health: A Comprehensive Review

**DOI:** 10.3390/biomedicines13102515

**Published:** 2025-10-15

**Authors:** Dragos Cozma, Cristina Tudoran, Cristina Văcărescu

**Affiliations:** 1 Institute of Cardiovascular Diseases Timisoara, 300310 Timisoara, Romania; dragos.cozma@umft.ro (D.C.); cristina.vacarescu@umft.ro (C.V.); 2 Cardiology Department, Victor Babeș University of Medicine and Pharmacy, 300041 Timisoara, Romania; 3 Research Center of the Institute of Cardiovascular Diseases Timisoara, 300310 Timisoara, Romania; 4 Department VII, Internal Medicine II, Discipline of Cardiology, Victor Babeș University of Medicine and Pharmacy, E. Murgu Square, Nr. 2, 300041 Timisoara, Romania; 5 County Emergency Hospital “Pius Brinzeu”, L. Rebreanu, Nr. 156, 300723 Timisoara, Romania; 6 Center of Molecular Research in Nephrology and Vascular Disease, Faculty of the Victor Babeș University of Medicine and Pharmacy, E. Murgu Square, Nr. 2, 300041 Timisoara, Romania

**Keywords:** statin-vitamin D interaction, cardiometabolic synergy, endothelial inflammation modulation

## Abstract

Statins are widely used lipid-lowering agents that significantly reduce cardiovascular morbidity and mortality by lowering LDL-cholesterol. Vitamin D, traditionally known for its skeletal role, is increasingly recognized for its cardiovascular relevance. This study aims to focus on the complex relationship between statins, vitamin D, and their impact on cardiovascular outcomes. Both molecules intersect metabolically at 7-dehydrocholesterol, raising interest in their potential interactions. While theoretical concerns exist about statins impairing vitamin D synthesis, clinical studies suggest a neutral or modestly positive effect on circulating 25(OH)D levels. Statins may increase vitamin D levels by inhibiting its catabolism (via CYP3A4) and enhancing absorption. Observational data also suggest synergy between statins and vitamin D in reducing inflammation, oxidative stress, endothelial dysfunction, and atherogenesis. Though large trials showed no benefit of vitamin D supplementation in cardiovascular event reduction among vitamin D–replete individuals, select subgroups (those deficient or with statin-induced myalgia) may benefit from targeted supplementation. Optimizing vitamin D status could improve statin tolerability and adherence, especially in high-risk populations such as the elderly or those with metabolic syndrome. This review highlights the complex interplay between statins and vitamin D and supports a personalized approach to supplementation in statin-treated patients, aiming to enhance cardiovascular protection without overtreatment.

## 1. Introduction

Statins are among the most commonly prescribed lipid-lowering agents worldwide and have been pivotal in the prevention and management of cardiovascular disease (CVD). By inhibiting HMG-CoA reductase, the rate-limiting enzyme in the cholesterol biosynthesis pathway, statins effectively reduce low-density lipoprotein cholesterol (LDL-C), thereby lowering the risk of myocardial infarction, stroke, and cardiovascular mortality. Their widespread use and proven efficacy have made them a cornerstone in both primary and secondary cardiovascular prevention strategies [1,2].

Vitamin D, a fat-soluble secosteroid hormone traditionally associated with bone health and calcium-phosphorus metabolism, has recently gained substantial attention for its broader biological effects, particularly in cardiovascular physiology and pathology. Observational studies have suggested associations between vitamin D deficiency (VDD) (serum 25-hydroxyvitamin D (25(OH)D) under 20 nmol/L), and an increased risk of hypertension, heart failure, atherosclerosis, and coronary artery disease (CAD) [3,4,5,6]. These findings have sparked interest in understanding the cardiovascular implications of vitamin D beyond its classical endocrine functions.

Interestingly, the biosynthetic pathways of cholesterol and vitamin D intersect at a common precursor, 7-dehydrocholesterol. This biochemical connection raises important questions about the potential impact of statin therapy on vitamin D metabolism. Since statins inhibit cholesterol synthesis, concerns have been raised that they might also inadvertently affect endogenous vitamin D production, particularly in populations already at risk for deficiency, such as the elderly or those with limited sun exposure [7,8].

Cardiometabolic synergy refers to the complementary effects of interventions that target overlapping pathways involved in both metabolic dysfunction and cardiovascular disease. Statins reduce LDL cholesterol and exert anti-inflammatory and endothelial-stabilizing effects, while vitamin D modulates similar pathways through RAAS suppression, cytokine inhibition, and endothelial function enhancement. Mechanistically, both agents may interact through shared enzymes such as CYP3A4 and transport proteins like SR-BI and NPC1L1, influencing vitamin D metabolism and absorption. Together, their combined use may enhance lipid control, attenuate inflammation, and improve vascular health—demonstrating a form of cardiometabolic synergy that supports integrated risk reduction, particularly in patients with coexisting dyslipidemia, insulin resistance, or vitamin D deficiency.

This review explores the complex and evolving relationship between statin therapy and vitamin D status. We will examine whether statin use significantly influences serum vitamin D concentrations and whether supplementation is warranted in statin-treated individuals [9,10]. Furthermore, we will delve into the role of vitamin D in cardiovascular disease, with a specific focus on its involvement in endothelial function, inflammation, atherosclerosis, and myocardial health [11]. Drawing upon recent studies on pathophysiological mechanisms, clinical trials, and large-scale meta-analyses, this review seeks to provide a comprehensive and evidence-based perspective on the interplay between statins, vitamin D, and cardiovascular outcomes.

## 2. Statins and Vitamin D: Mechanistic Interactions

### 2.1. Shared Precursors and Metabolic Pathways

Cholesterol and 25(OH)D, the main circulating form of vitamin D, are both synthesized through pathways that involve a common biochemical precursor—7-dehydrocholesterol (7-DHC). This molecule is a pivotal substrate located in the skin, where it undergoes photochemical conversion to previtamin D3 upon exposure to ultraviolet B (UVB) radiation, eventually forming vitamin D3. At the same time, 7-DHC is also a precursor for endogenous cholesterol synthesis through a series of enzymatic transformations. Statins exert their therapeutic effect by inhibiting HMG-CoA reductase, the rate-limiting enzyme in the mevalonate pathway responsible for cholesterol biosynthesis. By doing so, they reduce the production of cholesterol and its upstream intermediates, theoretically including 7-DHC. Based on this biochemical intersection, early theoretical concerns arose that statin therapy could inadvertently reduce the availability of 7-DHC for vitamin D synthesis, thereby impairing cutaneous production of vitamin D3 [7,12].

However, subsequent research and clinical evidence have shown that this interaction is far more complex than initially assumed. While the inhibition of upstream cholesterol synthesis could potentially reduce 7-DHC levels, compensatory mechanisms in the skin and liver, along with dietary and supplemental sources of vitamin D, appear to mitigate any significant clinical impact. Indeed, no well-conducted clinical trials to date have demonstrated a consistent decrease in serum 25(OH)D concentrations as a direct consequence of statin use [12,13].

### 2.2. Vitamin D Receptor Signaling and Cardiovascular Implications

Vitamin D exerts its physiological functions primarily through binding to the vitamin D receptor (VDR), a nuclear hormone receptor expressed in a wide range of tissues, including vascular endothelial cells, cardiomyocytes, smooth muscle cells, and immune cells. Upon activation by 1,25-dihydroxyvitamin D [1,25(OH)_2_D], VDR heterodimerizes with the retinoid X receptor (RXR) and binds to vitamin D response elements (VDREs) in the promoter regions of target genes, thereby regulating transcription [14,15]. This genomic signaling modulates key processes such as inhibition of pro-inflammatory cytokine production (e.g., TNF-α, IL-6), suppression of RAAS (renin–angiotensin–aldosterone system), enhancement of endothelial nitric oxide synthase (eNOS) expression, and inhibition of vascular smooth muscle proliferation and fibrosis [16,17]. Non-genomic actions have also been described, involving rapid activation of signaling pathways such as PI3K/Akt, MAPK, and intracellular calcium fluxes, which may contribute to vasodilatory and cardioprotective effects. These diverse actions of VDR signaling provide a mechanistic basis for the observed associations between vitamin D status and cardiovascular outcomes, supporting its role as a modifiable factor in atherosclerosis and heart failure [18].

### 2.3. Molecular Mediators and Transport Proteins Involved in Statin–Vitamin D Interactions

To further elucidate the biochemical intersection between statin therapy and vitamin D metabolism, it is critical to consider the specific enzymes and transport proteins that mediate the synthesis, activation, and catabolism of both molecules [19,20,21]. Statins exert their primary action through the inhibition of HMG-CoA reductase, the rate-limiting enzyme in the mevalonate pathway, thereby reducing downstream production of 7-dehydrocholesterol (7-DHC) and cholesterol. However, 7-DHC also serves as the substrate for UVB-mediated photoconversion in the skin to previtamin D3, which is subsequently isomerized into vitamin D3 (cholecalciferol). Vitamin D3 is then hydroxylated in the liver by cytochrome P450 2R1 (CYP2R1) to form 25-hydroxyvitamin D [25(OH)D], and further activated in the kidney via CYP27B1 into 1,25-dihydroxyvitamin D [1,25(OH)_2_D], the hormonally active form.

In parallel, CYP3A4, a major hepatic enzyme, is involved in the catabolism of both statins (particularly lipophilic agents like simvastatin and atorvastatin) and vitamin D metabolites. Competitive inhibition at CYP3A4 by statins may reduce the degradation of vitamin D, thereby increasing its circulating levels. In the gastrointestinal tract, vitamin D absorption is facilitated by sterol transporters, including Niemann–Pick C1-Like 1 (NPC1L1), Scavenger Receptor Class B type 1 (SR-B1), and Cluster of Differentiation 36 (CD36). Preclinical studies suggest that statins may modulate the expression or activity of these proteins, potentially enhancing vitamin D absorption from dietary or supplemental sources. Finally, CYP24A1, which catalyzes the inactivation of vitamin D metabolites, may also be indirectly influenced by statins through feedback mechanisms or modulation of inflammatory pathways, though this remains under investigation. These complex, overlapping molecular pathways underscore the need for further mechanistic studies to determine whether statins exert direct or tissue-specific regulatory effects on vitamin D homeostasis [20].

### 2.4. Influence on Vitamin D Synthesis and Metabolism

Several plausible mechanisms have been proposed to explain how statins might affect the metabolism and bioavailability of vitamin D, suggesting not only a neutral but potentially favorable effect on circulating vitamin D levels.

- CYP3A4 Competition: Many statins, particularly the lipophilic ones like atorvastatin and simvastatin, are metabolized in the liver via the cytochrome P450 3A4 (CYP3A4) enzyme system—the same enzymatic pathway responsible for catabolizing vitamin D into inactive metabolites. Competitive inhibition at this enzymatic site could slow the breakdown of vitamin D, thereby leading to higher circulating levels of 25(OH)D. This mechanism offers one explanation for why some studies have observed increased vitamin D levels in statin-treated individuals [22,23,24].
- Upregulation of Intestinal Absorption: Vitamin D is absorbed in the small intestine, a process facilitated by cholesterol transporters such as SR-BI (Scavenger Receptor Class B type I), CD36, and NPC1L1 (Niemann–Pick C1-like 1). These transporters are known to play a role in the absorption of lipid-soluble substances, including vitamin D. Statins may enhance the expression of these transporters, especially under experimental or high-dose conditions, potentially improving the efficiency of dietary vitamin D absorption [20].
- Alteration of 7-DHC Availability in the Skin: Despite reducing cholesterol synthesis overall, statins may cause a buildup of certain precursors, including 7-DHC, in specific tissues such as the skin. This could theoretically increase the substrate pool available for vitamin D3 production under UVB exposure, possibly explaining why some statin users exhibit improved bone health markers, such as increased bone mineral density. However, the evidence in human studies for this mechanism is still limited and mostly inferential [19,22].
- Vitamin D Receptor Activation: A more speculative but intriguing theory is that statins might directly or indirectly activate the vitamin D receptor (VDR). Since both statins and vitamin D exert overlapping cardioprotective effects—such as reducing inflammation, modulating immune responses, and improving endothelial function—it has been proposed by researchers like Bhattacharyya et al. that statins could mimic some of the actions of vitamin D by acting as partial agonists of VDR. Although compelling in concept, this idea remains largely hypothetical and has not yet been substantiated through direct clinical evidence [25].

Although cholesterol and vitamin D share a common metabolic origin in 7-DHC, and statins modulate this biosynthetic pathway, the net effect of statin therapy on vitamin D status appears to be neutral or mildly beneficial, rather than detrimental. Statins do not induce VDD at a population level, and in some cases, may even lead to modestly elevated 25(OH)D levels due to mechanisms like reduced catabolism and enhanced absorption [10,26].

**Figure 1:**
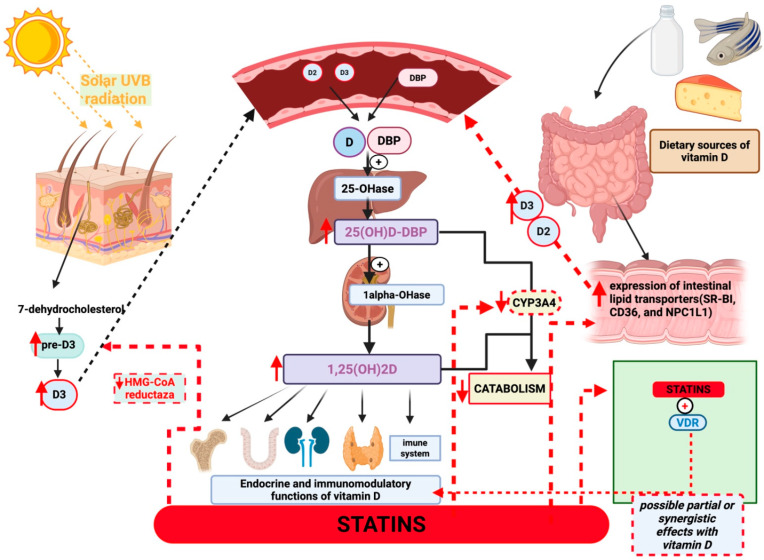
Statins and vitamin D: molecular links and shared pathways. D_3_—cholecalciferol (vitamin D_3_); D_2_—ergocalciferol (vitamin D_2_); DBP—vitamin D-binding protein; 25(OH)D—25-hydroxyvitamin D (calcidiol); 1,25(OH)_2_D—1,25-dihydroxyvitamin D (calcitriol); 25-OHase—hepatic 25-hydroxylase; 1α-OHase—renal 1α-hydroxylase; CYP3A4—cytochrome P450 3A4; HMG-CoA reductase—3-hydroxy-3-methylglutaryl-CoA reductase; SR-BI—scavenger receptor class B type I; CD36—cluster of differentiation 36; NPC1L1—niemann–pick C1-like 1 protein; VDR—vitamin D receptor.

Figure 1 illustrates the biochemical and physiological interactions between statin therapy and vitamin D metabolism, emphasizing their overlapping roles in cholesterol synthesis, inflammation modulation, endothelial function, and cardiovascular protection.

However, these effects likely vary depending on several factors, including: the specific statin used (lipophilic vs. hydrophilic), the dose and duration of therapy, individual genetic polymorphisms affecting metabolism, baseline vitamin D status, and concurrent dietary or environmental influences (e.g., sun exposure, nutritional intake) [27].

Understanding these nuanced interactions can help clinicians make more informed decisions about vitamin D monitoring and supplementation in patients undergoing long-term statin therapy.

In summary, while statins and vitamin D share metabolic pathways, statin therapy does not appear to induce VDD on a population level. On the contrary, pathophysiological pathways suggest statins might modestly increase vitamin D levels via reduced catabolism and enhanced absorption. The net effect likely varies among different statin drugs and patient contexts.

### 2.5. Combined Role of Statins and Vitamin D in Cardiovascular Risk Reduction: Synergy or Redundancy?

Overlap in Mechanisms of Action:

- Anti-Inflammatory Effects

Both statins and vitamin D exhibit potent anti-inflammatory properties, which are central to mitigating the pathogenesis of atherosclerosis. Statins reduce the production of pro-inflammatory cytokines such as IL-6, CRP, and TNF-α, and suppress macrophage activity within atherosclerotic plaques [28]. Vitamin D, through activation of the VDR, downregulates pro-inflammatory gene expression and modulates immune responses by promoting a more efficient anti-inflammatory cytokine response (e.g., increased IL-10) [29]. This dual anti-inflammatory action could theoretically stabilize atherosclerotic plaques more effectively than either agent alone, reducing the risk of plaque rupture and thrombosis.

Platelets may play a pivotal role in the pro-inflammatory and pro-aggregatory response which is one of the main pathophysiological mechanisms involved in atherosclerosis and atherogenesis. In his study, Ahmadi et al. [30] demonstrated that patients with CAD had significantly elevated platelet TGF-β1 levels, both latent and mature, compared to healthy subjects. Soluble TGF-β1 was also increased in patients with clinical atherosclerosis. This study determined significant correlations between mature/active TGF-β1 and platelet pro-inflammatory markers (P-selectin and CD40L) as well as common indicators of inflammation (CRP and ESR). Several studies highlighted an interaction between statins and platelets, with the implication of multiple mechanisms [31]. A recent study demonstrated that atorvastatin prevented TGF-β1-induced fibrogenesis in human ventricular fibroblasts, at least partially by inhibiting the Smad3 and MAPK signaling pathways, and therefore, this statin can be a promising drug for the treatment of myocardial fibrosis [27]. Through the activation of the VDR, vitamin D demonstrated antithrombotic effects in in vivo studies by upregulating platelet aggregation and by increasing endothelial NO synthesis. Other pathophysiological pathways consist of enhanced gene expression of antithrombin in the liver and of thrombomodulin in the liver, as well as in the aorta and kidney and in reducing tissue factor mRNA expression [32].

- Endothelial Protection

Endothelial dysfunction is a hallmark of cardiovascular disease and a target for both statins and vitamin D. Statins enhance endothelial nitric oxide synthase (eNOS) activity, improving nitric oxide (NO) bioavailability, which leads to vasodilation and vascular homeostasis [16,33]. Vitamin D also improves endothelial function by reducing oxidative stress, lowering endothelin-1 levels, and modulating NO production [34]. The concurrent use of statins and vitamin D might result in additive improvement in endothelial responsiveness, particularly in patients with diabetes or hypertension, where endothelial dysfunction is pronounced.

- Plaque Stabilization and Atheroprotection

Both agents have shown plaque-stabilizing effects in animal models and clinical studies. Statins promote fibrous cap thickening, reduce lipid core size, and inhibit matrix metalloproteinase activity, thereby reducing plaque vulnerability [35]. Vitamin D’s regulatory effects on macrophage activity, smooth muscle cell function, and vascular calcification may complement statins by slowing plaque progression and improving plaque composition [36].

- Modulation of Thrombogenesis

A pro-thrombotic state contributes to acute coronary syndromes. Statins reduce platelet aggregation and improve fibrinolysis [37]. VDD has been associated with a hypercoagulable state, including increased tissue factor expression and platelet activation. Supplementation may normalize hemostatic balance, especially in vitamin D-deficient individuals [5].

- Oxidative Stress Reduction

Oxidative stress promotes endothelial dysfunction, lipid peroxidation, and plaque instability. Statins decrease oxidative stress by downregulating NADPH oxidase and enhancing antioxidant enzymes [38]. Vitamin D exerts similar effects by reducing reactive oxygen species (ROS) and enhancing antioxidant defenses. Together, these agents may synergistically reduce oxidative burden, contributing to improved vascular integrity and myocardial protection [39]. Several studies indicate that the inhibition of ROS generation can have benefic effects on platelets [40,41].

Table 1 provides a summary of overlapping mechanisms by which statins and vitamin D exert cardiovascular protective effects, including anti-inflammatory actions, endothelial protection, plaque stabilization, modulation of thrombogenesis, and reduction in oxidative stress. Most references focus on human clinical or observational data, with a few incorporating animal or in vitro mechanistic findings (notably references [26,29]).

## 3. Changes in Vitamin D Levels in Statin Users: Clinical Evidence

Multiple clinical studies have explored how statin therapy impacts serum 25(OH)D concentrations, with mixed results:

- Rosuvastatin’s Notable Effect: Initial reports by Yavuz et al. observed a striking rise in 25(OH)D after initiating rosuvastatin. In an 8-week observational study, mean 25(OH)D increased from ~14 ng/mL to 36 ng/mL on rosuvastatin [7]. A subsequent randomized trial (STATIN-D) comparing rosuvastatin (10 mg) to fluvastatin found a similar tripling of 25(OH)D with rosuvastatin (11.8 to 35.2 ng/mL over 8 weeks), whereas fluvastatin (80 mg) showed no significant change. These findings led to the hypothesis of a rosuvastatin-specific effect. However, a critical re-analysis questioned the magnitude of this increase, suggesting that factors like seasonal sunlight variation might have contributed [19].
- Other Statins (Atorvastatin, Simvastatin, Lovastatin): Several studies suggest moderate increases in vitamin D with other statins. For example, an analysis by Pérez-Castrillón et al. in patients with CAD and baseline VDD found that 12 months of atorvastatin (10–80 mg/day) raised mean 25(OH)D from 16.4 to 18.8 ng/mL (*p* = 0.003) [47]. Similarly, small studies reported that lovastatin (20–80 mg) for 3 months led to higher 25(OH)D levels, and simvastatin therapy increased 25(OH)D and even active 1,25-dihydroxyvitamin D_3 levels [48]. Sathyapalan et al. observed a ~47% increase in 25(OH)D with 20 mg atorvastatin in patients with polycystic ovary syndrome [10]. On the other hand, Rejnmark et al. reported no significant change in vitamin D metabolites with simvastatin 40 mg over 8 weeks [49].
- Pravastatin and Fluvastatin: Hydrophilic statins like pravastatin have generally shown no effect on vitamin D levels. Trials of pravastatin (10–80 mg daily) demonstrated no difference in 25(OH)D between treated and control groups during a follow-up of 2 to 6 months [7]. As noted above, fluvastatin (another hydrophilic statin) did not alter vitamin D in the STATIN-D trial [7].
- Cross-Sectional Analyses: In a retrospective cross-sectional study of 384 patients, those on statins (mostly atorvastatin) had slightly higher serum vitamin D on average than matched non-users [22]. Short-term and medium-term statin users in that study had significantly higher 25(OH)D levels than non-users [34]. Notably, no group of statin-treated patients showed a decline in vitamin D compared to controls.

Meta-Analyses: When pooling data from various trials, the overall impact of statins on vitamin D appears modest. A meta-analysis of seven studies (including five RCTs) found no significant overall effect of statin therapy on plasma 25(OH)D levels [23]. Some discrepancies across studies may relate to differences in statin potency, lipophilicity, duration of therapy, baseline vitamin D status, and seasonal timing of measurements.

Table 2 summarizes the effects of various statins on serum 25(OH)D levels, presenting findings from different study designs—including observational studies, randomized controlled trials, and meta-analyses—while highlighting the type of statin used and reported changes in vitamin D concentrations.

Key Point: Current evidence does not support the notion that statins induce VDD. Statin therapy is often associated with stable or modestly increased vitamin D levels. For instance, rosuvastatin has been shown to increase serum 25(OH)D concentrations from approximately 14 ng/mL to 36 ng/mL over 8 weeks [7], while atorvastatin therapy led to an increase from 16.4 to 18.8 ng/mL over 12 months in patients with coronary artery disease [47].

## 4. Vitamin D Supplementation in Statin-Treated Patients

Given that statins do not typically cause vitamin D deficiency, is there a need for vitamin D supplementation in patients on statins? The answer depends on the clinical context:

- General Statin Users: For the average patient on statins, vitamin D supplementation should be guided by the same principles as in the general population—namely, treating vitamin D insufficiency or deficiency if present. There is no evidence that statin users benefit from extra vitamin D beyond the recommended dietary intake and sun exposure for bone health, unless they are deficient [5,7].
- Statin-Associated Muscle Symptoms: An important scenario is statin-induced myalgia or myopathy, which has been linked with low vitamin D status. Vitamin D deficiency can cause myalgias and muscle weakness on its own, and studies have observed that patients experiencing statin-associated muscle symptoms often have lower 25(OH)D levels than those who tolerate statins. In such cases, correcting the deficiency may improve symptoms. Notably, some patients with statin-associated myalgia and low vitamin D levels find relief after vitamin D repletion [43,50]. For example, one analysis reported that over 88% of previously statin-intolerant patients (with baseline 25(OH)D < 32 ng/mL) were able to tolerate statin rechallenge without myalgia after vitamin D supplementation corrected their deficiency. Another cohort found that 95% of statin-intolerant patients became free of muscle symptoms after 24 months of vitamin D repletion and statin retry [50,51]. While these are not randomized trials, they suggest that ensuring adequate vitamin D can mitigate statin myopathic side effects in susceptible individuals.
- Guidelines and Clinical Practice: Formal guidelines do not mandate vitamin D testing for all statin users, but many clinicians assess 25(OH)D in patients who develop muscle symptoms on statins. Ensuring a sufficient vitamin D status (e.g., >30 ng/mL) is a reasonable strategy to potentially reduce myopathic side effects and improve adherence to statin therapy. Vitamin D supplementation is inexpensive and safe in moderate doses, making it a practical consideration for patients at risk of deficiency or those with muscle complaints [52,53].

In summary, vitamin D supplementation is not universally required for every patient on a statin. However, identifying and treating VDD can be beneficial, particularly in those with statin-associated muscle complaints or other risk factors for low vitamin D. This targeted approach may enhance the tolerability and continued use of statins, indirectly supporting better cardiovascular outcomes.

### Guideline Recommendations for Vitamin D Testing and Supplementation

While formal guidelines do not currently mandate routine vitamin D screening for all statin users, several authoritative bodies do provide recommendations for vitamin D assessment in broader populations. For instance, the Endocrine Society advises screening individuals at high risk of deficiency, including older adults, those with osteoporosis, malabsorption syndromes, chronic kidney disease, or individuals presenting with unexplained musculoskeletal pain or weakness. Similarly, the National Osteoporosis Foundation and the Institute of Medicine recommend vitamin D supplementation for individuals with documented deficiency (<20 ng/mL), with target serum 25(OH)D levels generally set between 20 and 30 ng/mL depending on comorbidities and clinical context. In clinical practice, vitamin D testing is frequently considered in statin-treated patients who report myalgias or other muscle symptoms, as repletion may improve tolerance and reduce discontinuation risk. However, there is no current recommendation for universal screening or supplementation in statin users without specific risk factors or symptoms [54].

## 5. Vitamin D, Atherosclerosis, and Coronary Artery Disease

### 5.1. Vitamin D Levels and Cardiovascular Risk

A growing body of research has explored the link between vitamin D status and atherosclerotic cardiovascular disease. Observational studies consistently report that low 25(OH)D levels are associated with greater prevalence of cardiovascular risk factors (hypertension, diabetes, metabolic syndrome) and higher incidence of events. For instance, among male health professionals, those with VDD (<15 ng/mL) had approximately twice the risk of myocardial infarction compared to men with sufficient levels [4,55]. Similarly, in an observational cohort, each ~20 ng/mL increment in 25(OH)D was associated with a 33% reduction in the risk of fatal stroke [56]. A meta-analysis of more than 10 prospective studies (nearly 66,000 participants) found that individuals in the lowest vitamin D category had a 52% higher risk of total cardiovascular events compared to those with the highest vitamin D levels. This analysis showed a generally linear inverse relationship between 25(OH)D and cardiovascular risk down to about 60 nmol/L (~24 ng/mL), suggesting that deficiency is linked to elevated risk [57]. VDD has also been correlated with subclinical markers of atherosclerosis, such as increased carotid intima–media thickness and arterial stiffness, in some studies [58].

### 5.2. Pathophysiological Role of Vitamin D in Atherosclerosis

Biological plausibility for these associations is strong. VDRs are expressed in vascular endothelial cells, cardiomyocytes, vascular smooth muscle cells, and immune cells. Active vitamin D (1,25-dihydroxyvitamin D) exerts anti-inflammatory and immunomodulatory effects that could attenuate the chronic inflammation of atherosclerosis. Vitamin D also promotes endothelial function (by enhancing eNO availability and reducing oxidative stress) and may inhibit macrophage cholesterol uptake and foam cell formation, potentially stabilizing atherosclerotic plaques. Furthermore, vitamin D influences other cardiovascular factors: deficiency is linked to activation of the renin–angiotensin system (raising blood pressure), increased arterial stiffness, insulin resistance, and a pro-thrombotic profile—all of which can exacerbate atherosclerosis. Thus, adequate vitamin D levels might protect against atherosclerosis and its clinical consequences [11,59,60].

### 5.3. Vitamin D Supplementation and Cardiovascular Outcomes

Despite compelling observational data, randomized controlled trials have yet to conclusively demonstrate that vitamin D supplementation reduces cardiovascular events. Two large trials—VITAL (Vitamin D and Omega-3 Trial) and ViDA (Vitamin D Assessment Study)—tested moderate-to-high dose vitamin D in generally healthy, vitamin D–D-replete adults. Both reported no significant difference in major cardiovascular outcomes between the vitamin D and placebo groups [21,61]. In VITAL, for example, daily 2000 IU of cholecalciferol did not lower the incidence of myocardial infarction, stroke, or cardiovascular death over five years compared to placebo. These null results align with several prior smaller trials and meta-analyses, which collectively show no clear benefit of vitamin D supplementation for preventing heart attacks or strokes in unselected populations. It is important to note that the trial populations were largely vitamin D sufficient at baseline (mean 25(OH)D ~77 nmol/L in VITAL), leaving open the question of whether truly deficient patients might benefit [61]. Some subgroup analyses have hinted that participants with very low baseline vitamin D could have modest risk reductions with supplementation, but results have been inconsistent and not definitive.

### 5.4. Current Consensus

Based on available evidence, maintaining adequate vitamin D status is considered part of overall health optimization, but vitamin D alone is not a proven therapy to reduce established atherosclerosis or prevent CAD events. Current public health guidelines restrict vitamin D supplementation recommendations to bone health and fracture prevention, not cardiovascular protection. Nonetheless, given vitamin D’s low cost and safety, ongoing research is examining if certain high-risk groups (e.g., patients with both VDD and cardiovascular risk factors, or those with chronic kidney disease) might derive cardiovascular benefit from targeted vitamin D supplementation. For now, treating vitamin D deficiency in patients with atherosclerosis is reasonable for general health, while recognized preventive measures (statins, blood pressure control, diet, etc.) remain paramount for reducing cardiovascular risk [45,54].

### 5.5. Identifying Target Populations for Combined Therapy

Given that the benefits of vitamin D are more pronounced in deficient individuals, not all patients on statins will necessarily benefit from routine co-supplementation. Therefore, a targeted approach is key:

- Patients with confirmed vitamin D deficiency (<20 ng/mL): These individuals are most likely to benefit from supplementation, both for skeletal and possible cardiovascular protection. When treated with statins, addressing the deficiency could improve muscle tolerability and overall metabolic balance [18,21,43].
- Elderly or institutionalized patients: These populations frequently exhibit both low vitamin D levels and high cardiovascular risk. They are prime candidates for dual intervention [62].
- Patients with chronic inflammatory conditions (e.g., rheumatoid arthritis, metabolic syndrome): These conditions are associated with persistent low-grade inflammation, endothelial dysfunction, and oxidative stress—areas where vitamin D and statins may have complementary actions [63,64].

### 5.6. Optimizing Dosage and Monitoring

Statin Therapy: Prescribed according to current cardiovascular risk stratification guidelines: Consider using lipophilic statins (e.g., atorvastatin, simvastatin) in patients also receiving vitamin D, due to potential shared metabolic pathways (e.g., CYP3A4), but be cautious of interactions [23].

Vitamin D Supplementation: Follow standard dosing protocols for deficiency (e.g., 1000–2000 IU/day or weekly loading doses in severe cases). Monitor 25(OH)D levels periodically, especially in those with comorbidities such as chronic kidney disease or malabsorption and aim for a target level of >30 ng/mL, especially in patients with muscular symptoms or inflammation [54,65].

### 5.7. Preventive Cardiovascular Strategies

Incorporating both agents into multifactorial cardiovascular risk reduction plans may be especially useful in secondary prevention of myocardial infarction or stroke, especially in patients with low vitamin D level, in patients undergoing cardiac rehabilitation, where improvements in endothelial function and muscle performance may be enhanced by vitamin D status optimization and for diabetic or metabolic syndrome patients, where vitamin D may assist in insulin sensitivity and vascular health alongside statin therapy [66,67,68].

### 5.8. Multidisciplinary Integration

To implement this strategy effectively, primary care providers, cardiologists, endocrinologists, and clinical pharmacists should collaborate in identifying patients who may benefit from combination therapy. Integration into electronic health records (EHRs) could prompt clinicians to assess vitamin D status when prescribing statins, particularly for at-risk individuals, also patient education on lifestyle (diet, sun exposure) and medication adherence is critical to maximizing therapeutic outcomes.

## 6. Summary of Key Findings and Future Therapeutic Directions

### 6.1. Summary of Key Findings

- Statins and Vitamin D Levels: Contrary to initial expectations, statins do not appear to induce VDD. Some statins—notably rosuvastatin and atorvastatin—have even been associated with increased 25(OH)D levels in clinical studies. Proposed mechanisms include reduced vitamin D catabolism via CYP3A4 inhibition and enhanced intestinal vitamin D absorption via upregulated cholesterol transporters. Overall, no significant drop in vitamin D has been documented with statin use, and meta-analyses show a neutral net effect on 25(OH)D levels.
- Vitamin D Supplementation in Statin Users: Routine vitamin D supplementation solely because a patient is on statin therapy is not necessary if the patient is vitamin D replete. However, vitamin D status should be optimized in all individuals. In statin users who are vitamin D-deficient, repletion is advised—especially if they experience statin-associated myalgias. Evidence suggests correcting low vitamin D can alleviate statin-related muscle symptoms and improve tolerance to therapy. Ensuring adequate vitamin D may help patients continue statins, maximizing cardiovascular benefits.
- Vitamin D and Cardiovascular Disease: Low vitamin D levels have been consistently linked to a higher risk of atherosclerosis and adverse cardiovascular outcomes in observational studies. Vitamin D’s anti-inflammatory, endothelial, and metabolic effects provide a plausible explanation for these associations. However, randomized trials (in mostly vitamin D-sufficient populations) have not shown significant reductions in heart attacks or strokes with vitamin D supplementation. Therefore, while maintaining adequate vitamin D is important for overall health, it should complement—not replace—established cardiovascular prevention strategies. Further research is needed to determine if targeted vitamin D therapy in deficient, high-risk groups can improve cardiovascular outcomes.

### 6.2. Future Therapeutic Directions

- Fixed-dose combination therapy: Although not yet commercially available, the idea of combining statins with vitamin D in a single pill could be explored for specific populations.
- Personalized medicine approaches using genetic profiling (e.g., CYP3A4 polymorphisms, VDR gene variants) could help predict which patients will benefit most from combined therapy.

Adjunctive trials: Future randomized controlled trials should evaluate whether vitamin D supplementation in deficient statin users improves hard cardiovascular endpoints (e.g., MI, stroke, mortality), beyond symptom relief. Looking ahead, the integration of genetic and molecular profiling into clinical decision-making may enable more personalized strategies for combining statins and vitamin D. Specific genetic polymorphisms such as CYP3A4 variants, which influence the hepatic metabolism of lipophilic statins, and polymorphisms in the vitamin D receptor (VDR) gene have been implicated in interindividual variability in vitamin D responsiveness and could serve as predictors of treatment efficacy or adverse effects. Future clinical trials should investigate whether these genetic markers can stratify patients likely to derive enhanced cardiovascular or metabolic benefit from combined therapy. Additionally, randomized controlled trials should be designed to evaluate robust cardiovascular endpoints (e.g., myocardial infarction, stroke, cardiovascular death), alongside functional biomarkers such as endothelial reactivity, serum inflammatory mediators (e.g., IL-6, CRP), and statin tolerability in vitamin D-deficient individuals. These studies could clarify whether targeted co-supplementation improves clinical outcomes beyond those achievable by statins alone.

## 7. Limitations of the Study

An important methodological limitation of this review lies in the interpretation of subgroup analyses derived from the primary studies discussed. While subgroup findings can offer valuable preliminary insights into potential differential effects—such as those influenced by baseline vitamin D status, specific statin pharmacokinetics, or comorbid conditions—they are frequently exploratory in nature and lack adequate statistical power to support definitive conclusions. Moreover, many subgroup observations do not meet the threshold for statistical interaction testing and are not independently replicated across trials. As a result, they should be regarded as hypothesis-generating rather than confirmatory.

In the context of this manuscript, differential responses to statins or vitamin D supplementation across subgroups (e.g., vitamin D-deficient individuals or users of specific statin types) are reported primarily as observational trends. While mechanistically plausible, these findings must be interpreted with caution until validated by prospectively designed, adequately powered randomized controlled trials specifically addressing these interactions.

Also, it is important to acknowledge the potential for publication bias in the available literature. Trials or observational studies reporting favorable effects of vitamin D supplementation or statin–vitamin D interactions may be more likely to be published than studies showing neutral or null findings. This asymmetry could influence pooled or narrative assessments of the evidence, resulting in an overestimation of benefit or mechanistic plausibility.

Collectively, these limitations highlight the need for cautious interpretation of subgroup findings and published results, as well as the importance of future high-quality, prospective studies—including those with rigorous publication of null or negative outcomes—to more definitively characterize the interplay between statins, vitamin D, and cardiovascular risk.

## 8. Conclusions

Statin therapy does not appear to induce vitamin D deficiency and may modestly increase serum 25(OH)D concentrations, particularly with lipophilic statins such as rosuvastatin and atorvastatin. Although routine vitamin D supplementation is not indicated in all statin-treated individuals, targeted repletion in those with confirmed deficiency—especially in the context of statin-associated muscle symptoms—may enhance tolerability and therapeutic adherence. While low vitamin D is linked to higher cardiovascular risk, supplementation has not shown consistent benefit in reducing major cardiovascular events, highlighting the need for targeted research in high-risk, vitamin D-deficient populations.

## Figures and Tables

**Table 1:** Overlap of Statins and Vitamin D Mechanisms.

Mechanism/Pathway	Statin Mechanism	Vitamin D Mechanism	Representative Evidence (Study Type/Population)
Anti-inflammatory/immunomodulation	↓ IL-6, CRP, TNF-α; suppress macrophage activation in plaques	VDR activation; suppress pro-inflammatory genes; ↑ IL-10	Review of clinical and experimental studies on cardiovascular inflammation [1]
Endothelial protection/vasodilation	↑ eNOS activity, improved NO bioavailability	↓ oxidative stress; ↓ endothelin-1; modulate NO synthesis	Experimental endothelial cell studies; small vascular trials in humans [1]
Plaque stabilization/atheroprotection	Promote fibrous cap thickening; reduce lipid core; inhibit metalloproteinases	Regulate macrophages, smooth muscle cells; modulate vascular calcification	Histopathological plaque studies; cardiovascular biology reviews [1]
Modulation of thrombogenesis/coagulation	Reduce platelet aggregation; improve fibrinolysis	Vitamin D deficiency linked to hypercoagulability: ↑ tissue factor, ↑ platelet activation	Reviews on coagulation and vitamin D deficiency in CVD [1]
Oxidative stress reduction/redox balance	↓ NADPH oxidases; ↑ antioxidant enzymes (SOD, catalase)	Reduce ROS, enhance antioxidant defenses	In vivo and cross-sectional studies in hypertension and general populations [42]
Statin-associated muscle symptoms (SAMS)	—	Observational link: low 25(OH)D associated with increased SAMS risk	Meta-analysis of observational studies (*n* ≈ 2400) [43]; RCT (VITAL sub-study, *n* = 2083) found no difference [43]
Adherence/persistence with statin therapy	—	Monthly vitamin D improved persistence (HR = 1.15, *p* = 0.02) but not adherence	Secondary analysis of long-term statin users in randomized vitamin D supplementation trial [44]
Cardiovascular outcomes (MACE)	Robust reduction in major adverse cardiovascular events across many RCTs	No consistent reduction in MACE; large RCTs and meta-analyses generally neutral	Meta-analysis of 14 RCTs (*n* ≈ 80,500, age 50–74) showed no significant benefit [21,45] DAYLIGHT trial no biomarker improvement [46]

↑—increased value, ↓—decreased value.

**Table 2:** Effects of stations on Vitamin D levels.

Statin(s) Studied	Study Design	Effect on Vitamin D Levels	Remarks/Limitations
Rosuvastatin	Observational study. [34]	Substantial increase (~14 to 36 ng/mL over 8 weeks)	Possible confounding, small sample
Rosuvastatin vs. Fluvastatin	Randomized controlled trial (STATIN-D) [35]	Rosuvastatin tripled 25(OH)D levels; Fluvastatin showed no change	Head-to-head comparison, limited duration
Atorvastatin	Longitudinal study in CAD patients [37]	Moderate increase (from 16.4 to 18.8 ng/mL over 12 months)	Slow change; possible seasonal/lifestyle influences
Lovastatin	Small clinical study [38]	Reported increase after 3 months	Very small sample, uncontrolled
Simvastatin	Small studies and one trial [40]	Reported increase in 25(OH)D and 1,25-dihydroxyvitamin D_3_; some studies show no change	Heterogeneous designs and populations
Atorvastatin (Cross-sectional)	Retrospective cross-sectional study (citation [43])	Slightly higher vitamin D levels in statin users vs. non-users	Association only; cannot prove causation
Pravastatin	Multiple clinical trials [41]	No significant difference compared to control over 2–6 months	Negative/null results in short-term trials
Fluvastatin	Randomized trial (STATIN-D) [42]	No significant change	Similarly to control arm in STATIN-D trial
Various (Meta-analysis)	Meta-analysis of 7 studies including 5 RCTs [36]	Overall modest or no significant effect	

## Data Availability

No new data were created or analyzed in this study. Data sharing is not applicable to this article.

## Abbreviations

The following abbreviations are used in this manuscript:

CVD	Cardiovascular Disease
LDL-C	Low-Density Lipoprotein Cholesterol
CAD	Coronary Artery Disease
HMG-CoA	3-Hydroxy-3-Methylglutaryl-Coenzyme A
25(OH)D	25-Hydroxyvitamin D
7-DHC	7-Dehydrocholesterol
UVB	Ultraviolet B
VDR	Vitamin D Receptor
CYP3A4	Cytochrome P450 3A4
SR-BI	Scavenger Receptor Class B type I
NPC1L1	Niemann–Pick C1-like 1
IL-6	Interleukin 6
CRP	C-Reactive Protein
TNF-Î±	Tumor Necrosis Factor Alpha
IL-10	Interleukin 10
eNOS	Endothelial Nitric Oxide Synthase
NO	Nitric Oxide
ROS	Reactive Oxygen Species
RCT	Randomized Controlled Trial
IU	International Units
MI	Myocardial Infarction
EHR	Electronic Health Record
